# Draft genome sequence data of methanotrophic *Methylovulum psychrotolerans* strain S1L and *Methylomonas paludis* strain S2AM isolated from hypoxic water column layers of boreal lakes

**DOI:** 10.1016/j.dib.2021.107364

**Published:** 2021-09-12

**Authors:** Antti J. Rissanen, Rahul Mangayil, Mette Marianne Svenning, Ramita Khanongnuch

**Affiliations:** a Faculty of Engineering and Natural Sciences, Tampere University, P.O. Box 541, Tampere 33014, Finland; b Department of Arctic and Marine Biology, UiT, The Arctic University of Norway, Tromsø 9037, Norway

**Keywords:** Methanotroph, *Methylovulum*, *Methylomonas*, Genome, Methane oxidation, Lake, Hypoxic, Microaerobic

## Abstract

Methanotrophic bacteria inhabit a wide range of natural (e.g. wetlands, lakes and soils) and anthropogenic (e.g. wastewater treatment plants and landfills) environments. They play a crucial role in mitigating atmospheric emissions of the greenhouse gas methane. There is also a growing interest in applying methanotrophs in the bioconversion of biogas - and natural gas - methane into value-added products (e.g. chemicals and single-cell protein). Hence, isolation and genome sequencing of methanotrophic bacteria is needed to provide important data on their functional capabilities. Here, we describe the *de novo* assembled draft genome sequences of *Methylovulum psychrotolerans* strain S1L isolated from hypoxic water column layer of boreal Lake Lovojärvi (Southern Finland), comprising total of 5090628 bp in 11 contigs with G+C – content of 50.9% and containing 4554 coding sequences. The draft genome of strain S1L represents the first published genome of *M. psychrotolerans* strain isolated from lake ecosystems. In addition, we present the genome sequence of *Methylomonas paludis* strain S2AM, isolated from water column of boreal Lake Alinen Mustajärvi (Southern Finland), comprising 3673651 bp in 1 contig with G+C – content of 48.2% and 3294 coding sequences. The draft genome of strain S2AM represents the first published genome of *M. paludis*. The preliminary genome annotation analysis of both S1L and S2AM identified genes encoding oxidation of methane, methanol, formaldehyde and formate, assimilation of carbon, ammonium and nitrate, N_2_ fixation, as well as various enzymes enabling the survival in hypoxic conditions, i.e. high-affinity oxidase, hemerythrins, fermentation enzymes (for production of acetate, succinate and H_2_) and respiratory nitrite reductases. The draft genomes have been deposited at GenBank under the accession JAGVVN000000000 for S1L and CP073754 for S2AM.

## Graphical abstract

**Figure:**
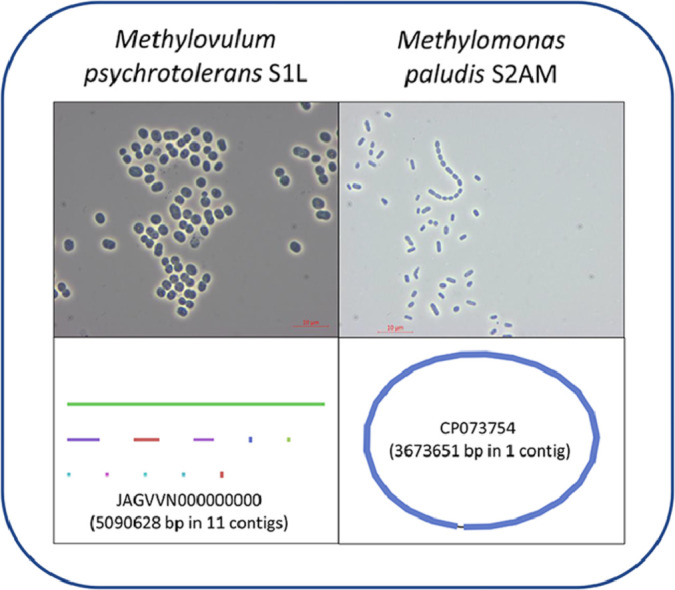

**Specifications Table**

**Table:** 

Subject	Biological sciences
Specific subject area	Bacterial Genomics, Applied Microbiology and Biotechnology, Biogeochemistry, Environmental Microbiology
Type of data	Genomic sequence Table Figure
How data were acquired	Whole genome sequencing using Illumina NovaSeq 6000 platform for short reads and Sequel SMRT Cell 1M v2 for long reads.
Data format	Raw Assembled/analyzed
Parameters for data collection	Strains S1L and S2AM were isolated from hypoxic boreal lake water samples. Their genomic DNA was isolated. Raw sequence reads were generated using Illumina NovaSeq 6000 (short reads) and Sequel SMRT Cell 1M v2 (long reads). Data was filtered (Trimmomatic), de novo assembled (Unicycler) and annotated (NCBI prokaryotic genome annotation pipeline, KofamKoala, PhyloPhlAn).
Description of data collection	Strain isolation, DNA extraction, sequencing library preparation, short and long read sequencing, raw data filtering, draft de novo assembly and annotation procedures.
Data source location	Institution: Tampere University City/Town/Region: Tampere Country: Finland Isolation sources: Strain S1L: Lake Lovojärvi (61° 04′N, 25° 02′E) Strain S2AM: Lake Alinen Mustajärvi (61° 12′ N, 25° 06′ E)
Data accessibility	Repository: The assembled whole-genome shotgun data (.fasta) has been deposited at GenBank under the accession numbers JAGVVN000000000 for S1L (https://www.ncbi.nlm.nih.gov/nuccore/JAGVVN000000000) and CP073754 for S2AM (https://www.ncbi.nlm.nih.gov/nuccore/CP073754). The raw sequence data (.fastq) have been deposited in the SRA database under the Bioproject PRJNA726368 (https://www.ncbi.nlm.nih.gov/sra/PRJNA726368) and Biosample SAMN18928743 for S1L and Bioproject PRJNA724036 (https://www.ncbi.nlm.nih.gov/sra/PRJNA724036) and Biosample SAMN18839544 for S2AM. The 16S rRNA gene sequences (.fasta) are deposited at GenBank under accession numbers MZ052218 for S1L and MZ052219 for S2AM.

**Value of the Data**

• Draft genome sequences of *Methylovulum psychrotolerans* strain S1L and *Methylomonas paludis* strain S2AM provide fundamental knowledge on the functional potential of methanotrophs mitigating methane emissions from natural freshwater ecosystems and give insights into their biotechnological applicability
• This data is beneficial for researchers in biogeochemistry, environmental microbiology, biotechnology and circular economy
• This data can be used in predicting the function of methanotrophs in natural ecosystems under variating physicochemical conditions as well as in developing methane-based bioproduct platforms

## 1 Data Description

**Fig. 1:**
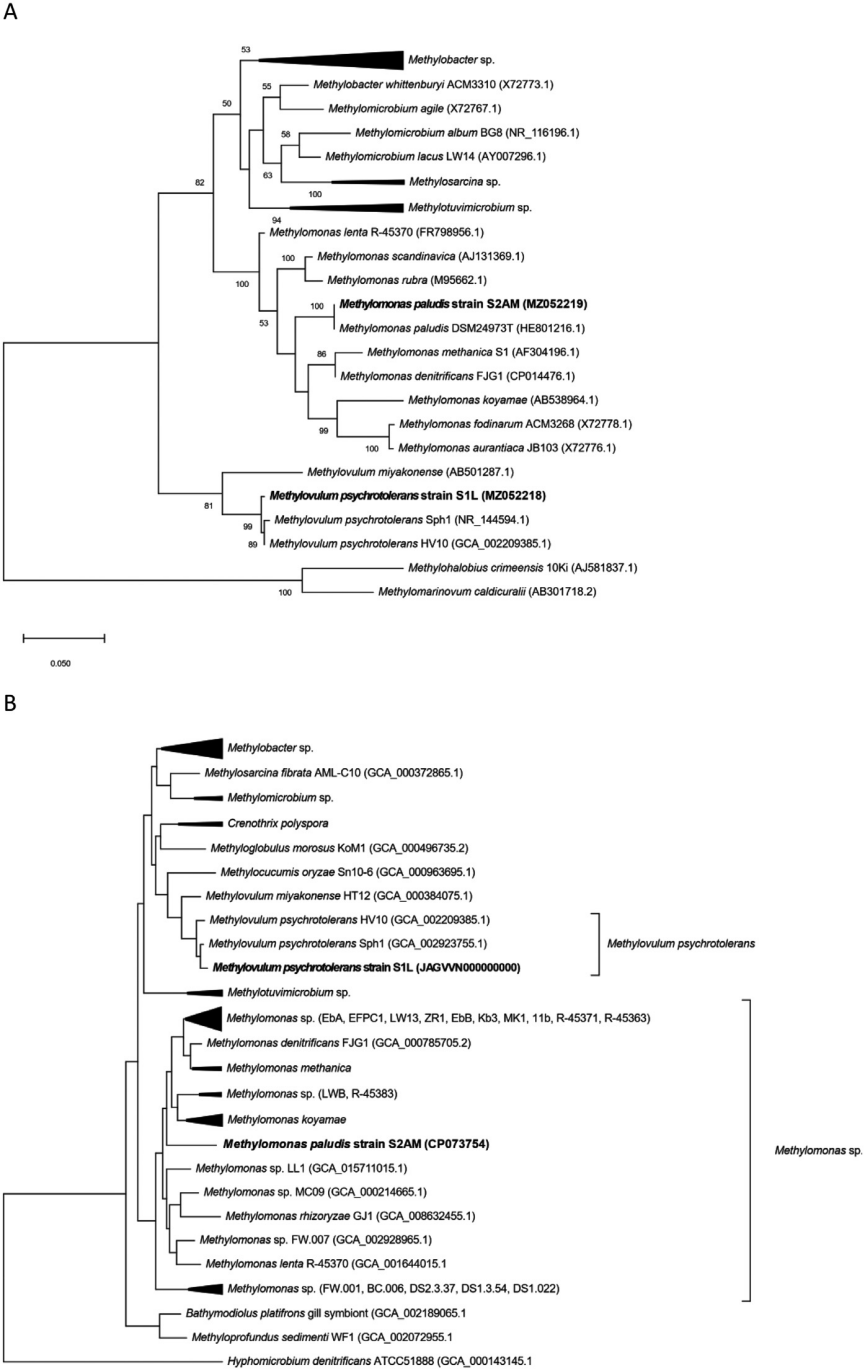
(A) Phylogenetic tree based on 16S rRNA genes and (B) Genome-wide phylogenomic tree (PhyloPhlAn). Strains S1L and S2AM are highlighted in bold.

Methanotrophic bacteria are widely distributed in natural (wetlands, lakes, oceans, soils) and anthropogenic (wastewater treatment plants, landfills) methane-producing ecosystems [1,2]. Two methanotrophic strains, S1L and S2AM, were isolated from hypoxic water column layer of boreal Lake Lovojärvi and Lake Alinen Mustajärvi, respectively. Based on the 16S rRNA gene sequencing and phylogenetic tree analysis, we classified S1L and S2AM as representatives of *Methylovulum psychrotolerans* and *Methylomonas paludis*, respectively (see 16S rRNA-gene based phylogenetic tree in Fig. 1A). We chose strains S1L and S2AM for draft genome sequencing to identify their functional potential because

1) methanotrophs are important mitigators of atmospheric methane emissions,
2) methanotrophs provide platforms for bioconversion of biogas- and natural gas- methane to value-added bioproducts [1,3], and
3) there are no previously published genomes of *M. paludis*, and for *M. psychrotolerans* no genomes of lake strains exist, as the previously published genomes are from strains Sph1 and HV10-M2 isolated from cold methane seep and soil ecosystems, respectively.

The full statistics of *de novo* assemblies and genome characteristics of strains S1L and S2AM are reported in Table 1. The draft genome of S1L consisted of 11 contigs, with 5090628 bp in total length, G+C content of 50.9%, 4554 coding sequences, 9 rRNAs and 46 tRNA genes. In accordance with 16S rRNA gene – based analyses (Fig. 1A), phylogenomic tree analysis (see Fig. 1B) as well as average nucleotide identities higher than the 95% - identity level used for species-level delineation, i.e. 97.4% to *M. psychrotolerans* Sph1 (type strain) and 97.3% to *M. psychrotolerans* HV10-M2, confirmed that strain S1L is a representative of *M. psychrotolerans*.

**Table 1:** Statistics of *de novo* assemblies and genome characteristics of strains S1L and S2AM.

Feature/Strain	Strain S1L	Strain S2AM
Total sequence length (bp)	5090628	3673651
Number of contigs	11	1
N50 (bp)	3779276	3673651
G + C - content (%):	50.9	48.2
Number of coding sequences	4554	3294
Number of 5S, 16S and 23S rRNA genes	3 (5S), 3 (16S), 3 (23S)	3 (5S), 3 (16S), 3 (23S)
Number of tRNA genes	46	44
CRISPR Arrays	4	4

The draft genome of S2AM consisted of 1 contig, with 3673651 bp in length, G+C content of 48.2%, 3294 coding sequences, 9 rRNA and 44 tRNA genes (Table 1). The deduced amino acid sequences of the database-deposited genes of the type strain *M. paludis* MG30(T) (DSM 24973) [4], i.e. pmoA (CCH22593.1), mxaF (CCH22594.1) and nifH (CCH22595.1), were 100% identical to the deduced amino acid sequences of the respective genes in the genome of strain S2AM, which confirms the results of 16S rRNA gene analyses on the species-level classification of strain S2AM (Fig. 1A). Average nucleotide identities of genome of S2AM to genomes of other strains of *Methylomonas* were not reported by FastANI program confirming that they were all <80% [5]. The genome of S2AM also formed a separate species-level branch in the phylogenomic tree (Fig. 1B).

Both S1L and S2AM encoded particulate methane monooxygenase operon (pmoCAB) for conversion of methane to methanol. In addition, both strains encoded the pxm operon (pxmABC), i.e. a copper membrane monooxygenase of unknown function [6], while genes coding for the soluble methane monooxygenase (mmoXYZBCD) were not found in either of the strains. For the conversion of methanol to formaldehyde, the strains coded for both calcium- (mxaFJGIACKLD) and lanthanide-dependent (xoxF) methanol dehydrogenases. Genes involved in tetrahydromethanopterin (H4MTP) - and tetrahydrofolate (H4-folate) - linked C1 transfer (i.e. formaldehyde oxidation), as well as in formate oxidation were also identified in both strains. Genes for a RuMP pathway for carbon (formaldehyde) assimilation and the oxidative TCA cycle were also present in both strains.

The genomes of both strains also included genes encoding nitrogen fixation (nitrogenase, nifDKH) and assimilation of ammonium (ammonium transporters, alanine dehydrogenase, glutamine synthetase/glutamate synthase) and nitrate (nitrate/nitrite transporters, nitrate reductase nasA and nitrite reductase nirBD). In accordance with their origin from hypoxic lake waters, genes encoding enzymes enabling for the survival in hypoxic conditions were found in both strains, i.e. high-affinity oxidase (cytochrome bd-I ubiquinol oxidase), oxygen-binding hemerythrins, nirS-type dissimilatory nitrite reductase as well as various fermentation enzymes, i.e. succinate dehydrogenase (sdhABCD) for conversion of fumarate to succinate, acetate kinase (ackA) for conversion of acetyl phosphate to acetate and hydrogen dehydrogenase (hoxFGHY) for conversion of H+ to H_2_ [7], [8], [9].

## 2 Experimental Design, Materials and Methods

### 2.1 Isolation of strains and DNA extraction

Strain S1L and S2AM were isolated from water samples, which were collected using a 2 dm^3^ Limnos water sampler (length 30 cm) (Limnos Ltd., Turku, Finland) at 3.25 m depth of Lake Lovojärvi (61° 04′N, 25° 02′E; area = 0.054 km^2^, max. depth = 17.5 m) and at 4.5 m depth of Lake Alinen Mustajärvi (61° 12′N, 25° 06′E; area = 0.007 km^2^, max. depth = 6.5 m), located in Southern Finland, in 3rd of September 2019. The lakes were sampled at their deepest points, where the water was also vertically stratified with respect to O_2_ and temperature (T) as determined using YSI ProODO (optical dissolved oxygen) field meter (Yellow Springs Instruments, Yellow Springs, OH, USA). At the time of sampling, T, O_2_ and pH of the sampling layer were 12 °C, 12 µmol L^−1^ and 6.83, respectively in Lake Lovojärvi (strain S1L), and 6.3 °C, 10 µmol L^−1^, 5.76, respectively, in Lake Alinen Mustajärvi (strain S2AM). Hence, the strains were isolated from hypoxic water layers.

The strain S1L was enriched and purified using nitrate mineral salts (NMS) medium (DSMZ 921 medium; initial pH ∼6.80) supplemented with 1 µM lanthanum (LaCl_3_). The 1/10 dilution of NMS medium (dNMS) with 0.1 µM lanthanum was used for enrichment and purification of the strain S2AM. Initially, lake water samples were incubated in a serum bottle containing NMS or dNMS liquid media and 20% methane:80% air in headspace. After three or four subculturing in liquid media, the enriched lake cultures were streaked on NMS or dNMS agar media (1.5% noble agar) and incubated in an air-tight chamber with 20% methane:80% air in headspace for 14–30 days. Colonies were observed under stereo microscopy and picked and re-streaked until a single type of a colony was obtained. Culture purity was determined using a light microscope, and by confirming absence of growth in nutrient-rich medium and in NMS medium without CH_4_.

Genomic DNA was extracted using GeneJET Genomic DNA Purification Kit and quantified using a Qubit 2.0 Fluorometer and a dsDNA HS Assay Kit (Thermo Fisher Scientific, Waltham, MA, USA). Using the identification service offered by Macrogen (Amsterdam, Netherlands), the 16S rRNA genes of S1L and S2AM were amplified from the DNA using primers 27F (AGAGTTTGATCMTGGCTCAG) and 1492R (TACGGYTACCTTGTTACGACTT) and sequenced with primer pairs 785F (GGATTAGATACCCTGGTA) and 907R (CCGTCAATTCMTTTRAGTTT). 16S rRNA gene – based phylogenetic tree was done using the maximum likelihood algorithm (generalized time reversible model) with 100 bootstraps in Mega X [10].

### 2.2 Genome sequencing and analysis

Library preparation and sequencing for long and short reads was done by Novogene Co. Ltd. (Beijing, China). For long reads, the 10kb SMRTbell library was prepared using SMRTbell Template Prep Kit 1.0-SPv3 (catalog number 100-991-900) (Pacific Biosciences, Menlo Park, CA, USA). In library preparation, the qualified high-molecular weight DNA were fragmented to approx. 10 kb, followed by damage repair, end repair and adapter ligation. Afterwards, size selection was performed by Size-Selection System. The SMRTbell-Polymerase Complex was prepared using Sequel Binding Kit 2.0 and sequenced on Sequel SMRT Cell 1M v2 (Pacific Biosciences). Short read libraries were prepared using NEBNext® Ultra™ DNA Library Prep Kit for Illumina® (catalog number E7370L) (insert size 350 bp) (New England Biolabs, Ipswich, MA, USA) and the sequencing was done on Illumina NovaSeq 6000 platform (paired-end 150 bp) (Illumina, San Diego, CA, USA).

The long-read data was filtered using SMRTlink software (parameters: minLength=0, minReadScore=0.8), which resulted in libraries consisting of 190492 reads (1.554 Gb) and 168230 reads (1.628 Gb), with an average length (N50) of 8160 (9144) and 9678 (11070) bases for strains S1L and S2AM, respectively. Short read library sizes were 8880484 reads (1.3 Gb) and 7543586 reads (1.1 Gb) for S1L and S2AM, respectively. Trimmomatic (version 0.39) was used to remove low quality reads and reads containing adapters from short read libraries by applying parameters: ILLUMINACLIP:adapters.fasta:2:30:10 LEADING:3 TRAILING:3 SLIDINGWINDOW:4:15 MINLEN:36, for strain S1L, and parameters: ILLUMINACLIP:adapters.fasta:2:30:10 LEADING:3 TRAILING:3 SLIDINGWINDOW:4:20 MINLEN:50, for strain S2AM [11]. The genomes were assembled *de novo* using hybrid assembly strategy in Unicycler (version 0.4.8) with default parameters and “–mode normal” [12]. The genomes were annotated using the NCBI prokaryotic genome annotation pipeline [13]. The protein coding genes were also predicted using Prodigal (version 2.6.3) [14] and functionally annotated to KEGG orthology using KofamKoala (https://www.genome.jp/tools/kofamkoala/) [15]. Phylogenomic trees were built using PhyloPhlAn (version 3.0.58) with the PhyloPhlAn database (400 universal marker genes) and “–diversity low” - argument for “species- and strain-level phylogenies” [16]. Average nucleotide identities with reference genomes were computed using fastANI (version 1.32) [5].

## CRediT Author Statement

**Antti J. Rissanen:** Conceptualization, Formal analysis, Investigation, Writing – original draft, Visualization, Project administration, Funding acquisition; **Rahul Mangayil:** Conceptualization, Writing – review & editing, Formal analysis, Investigation, Funding acquisition; **Mette Marianne Svenning:** Supervision, Methodology, Writing – review & editing; **Ramita Khanongnuch:** Conceptualization, Writing – review & editing, Formal analysis, Investigation.

## Declaration of Competing Interest

The authors declare that they have no known competing financial interests or personal relationships which have, or could be perceived to have, influenced the work reported in this article.
